# Supplementary figures and images for: Lactobacillus plantarum Zhang-LL Inhibits Colitis-Related Tumorigenesis by Regulating Arachidonic Acid Metabolism and CD22-Mediated B-Cell Receptor Regulation
Source: Nutrients. 2023 Oct 25;15(21):4512. doi: 10.3390/nu15214512 (PMC10648432; doi:10.3390/nu15214512)

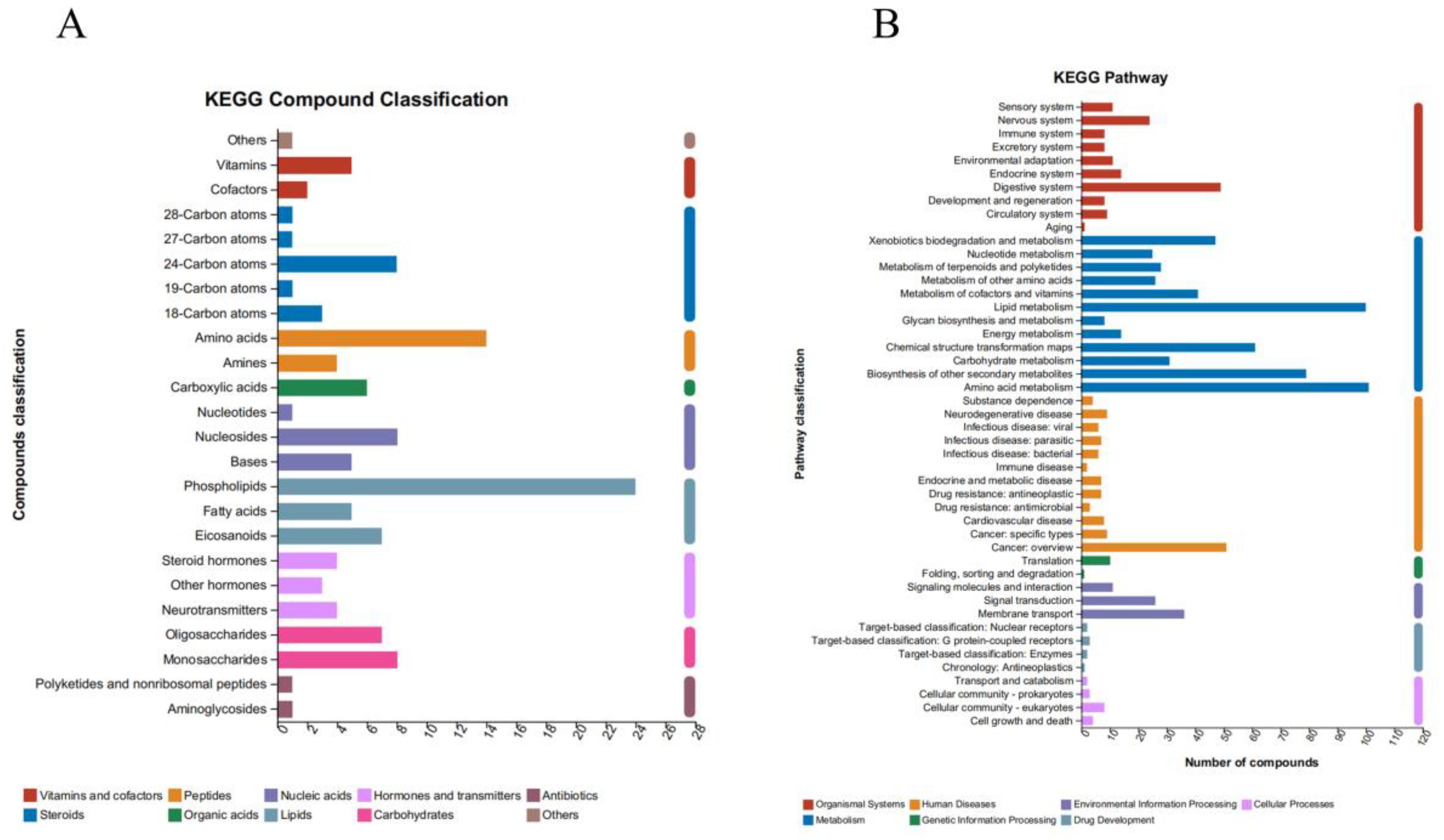

Supplement: Supplementary file 1 [file nutrients-15-04512-s001.zip › Fig S2.tif]
